# Mitophagy in Brain Injuries: Mechanisms, Roles, and Therapeutic Potential

**DOI:** 10.1007/s12035-025-04936-z

**Published:** 2025-04-16

**Authors:** Jiayu Tian, Yanna Mao, Dandan Liu, Tao Li, Yafeng Wang, Changlian Zhu

**Affiliations:** 1 Henan Neurodevelopment Engineering Research Center for Children, Children’s Hospital Affiliated to Zhengzhou University, Henan Children’s Hospital, Zhengzhou Children’s Hospital, Zhengzhou, 450018 China; 2 Department of Hematology and Oncology, Children’s Hospital Affiliated to Zhengzhou University, Henan Children’s Hospital, Zhengzhou Children’s Hospital, Zhengzhou, 450018 China; 3 Department of Electrocardiogram, Children’s Hospital Affiliated to Zhengzhou University, Henan Children’s Hospital, Zhengzhou Children’s Hospital, Zhengzhou, 450018 China; 4 Henan Key Laboratory of Child Brain Injury and Henan Pediatric Clinical Research Center, Institute of Neuroscienceand , Third Affiliated Hospital of Zhengzhou University, Zhengzhou, 450052 China; 5 Center for Brain Repair and Rehabilitation, Institute of Neuroscience and Physiology, Sahlgrenska Academy, University of Gothenburg, 40530 Göteborg, Sweden

**Keywords:** Mitophagy, Brain injury, Mitophagy regulation, Cell death, Therapeutic strategy

## Abstract

Mitophagy is an intracellular degradation pathway crucial for clearing damaged or dysfunctional mitochondria, thereby maintaining cellular homeostasis and responding to various brain injuries. By promptly removing damaged mitochondria, mitophagy protects cells from further harm and support cellular repair and recovery after injury. In different types of brain injury, mitophagy plays complex and critical roles, from regulating the balance between cell death and survival to influencing neurological recovery. This review aims to deeply explore the role and mechanism of mitophagy in the context of brain injuries and uncover how mitophagy regulates the brain response to injury and its potential therapeutic significance. It emphasizes mitophagy’s potential in treating brain injuries, including reducing cell damage, promoting cell recovery, and improving neurological function, thus opening new perspectives and directions for future research and clinical applications.

## Introduction

The term “brain injury” refers to a range of neurological conditions with significant impacts on human health. The injuries are characterized by a variety of causes and mechanisms, including cellular hypoxia, inflammatory response, oxidative stress, and the activation of cell death pathways [1–3]. These mechanisms lead to the damage or death of neural cells, thereby affecting brain function. The consequences of brain injury on an individual’s health can be transient or long lasting, potentially resulting in cognitive impairment, reduced motor capacity, and changes in mood and behavior. These outcomes can profoundly affect the quality of life and the ability to engage fully in society [4].

Mitochondria, as the energy center of cells, not only provide the energy necessary for life but also regulate critical life processes such as cell growth, division, and death [5, 6]. Abnormal mitochondrial function, such as energy metabolism disorders, excessive production of reactive oxygen species (ROS), and activation of apoptosis signals, can severely impact cell operation and survival [7].

Mitophagy is a distinct form of autophagy that occurs within cells. It is responsible for the removal of damaged or dysfunctional mitochondria, thereby maintaining the intracellular mitochondrial mass and ensuring functional balance [8, 9]. Recent studies have demonstrated the critical function of mitochondrial autophagy in the pathological process associated with brain injury [10, 11]. Following brain injury, mitochondrial dysfunction results in inadequate energy supply and elevated oxidative stress, which subsequently induces mitophagy as a self-protective mechanism. However, excessive activation of mitophagy can have deleterious effects on neurons, potentially resulting in cellular dysfunction or even death [12, 13]. As a result, the precise role of mitophagy in various forms of brain injury and the underlying regulatory mechanisms remain elusive, impeding a comprehensive understanding of brain injury treatment mechanisms and the development of therapeutic strategies. Therefore, a comprehensive investigation of the role of mitophagy in brain injury is crucial for elucidating the molecular mechanisms underlying brain injury and for developing novel therapeutic strategies.

### Mitochondria and Brain Injury

Mitochondria, essential double-membrane-bound organelles in eukaryotic cells, function as the primary sites of cellular energy production through oxidative phosphorylation. In the nervous systems, these dynamic organelles play crucial roles beyond adenosine triphosphate (ATP) synthesis. The regulate calcium ion buffering to maintain ionic homeostasis, support neurotransmitter biosynthesis and release, and modulate apoptotic signaling cascades in both neuronal and glial cell populations [14]. Recent studies highlight mitochondrial dysfunction as a key factor in brain injury and neurological diseases, including ischemic stroke, traumatic brain injury, and neurodegenerative diseases [15–17].

Mitochondria are vital for brain function. They supply the brain’s high energy demand, producing ATP to sustain neuronal and glial activity [18, 19]. Additionally, they help regulate intracellular calcium homeostasis, preventing toxic overload that can impair synaptic plasticity and neuronal excitability [20, 21]. Mitochondria also contribute to neurotransmitter synthesis, including glutamate, GABA, and dopamine, making their dysfunction detrimental to neural signaling and brain function [22–24].

Mitochondria play a dual role in brain injury. On one hand, ischemia and trauma can damage mitochondria, disrupting mitochondrial membrane integrity, impairing ATP production, and triggering apoptotic pathways through mitochondrial permeability transition pore (mPTP) opening and cytochrome *c* and apoptosis inducing factor release [25–27]. On the other hand, mitochondria contribute to recovery through dynamic processes such as fission, fusion, and mitophagy, which help clear damaged mitochondria and maintain network integrity [28, 29].

### Autophagy

Autophagy is a vital intracellular degradation and recycling system that maintains cellular homeostasis, regulates stress responses, and balances cell survival and cell death [30]. It has three main types: macroautophagy, microautophagy, and chaperone-mediated autophagy (CMA). Macroautophagy is the most studied form and involves the formation of autophagosomes that encapsulate damaged proteins and organelles, which then fuse with lysosomes for degradation. Microautophagy directly engulfs cytoplasmic components via lysosomal invagination, which CMA selectively degrades specific proteins transported into lysosomes by chaperones [31].

Autophagy follows key steps: (1) initiation, triggered by ULK1 in response to stress; (2) nucleation, where Beclin- 1 and VPS34 help form the autophagosome precursor; (3) elongation and maturation, assisted by ATG proteins; and (4) fusion and degradation, where lysosomal enzymes break down contents for cellular reuse [32]. The process enables cells to adapt to stress, remove damaged organelles, and maintain metabolic balance [33, 34]. Dysregulated autophagy is linked to neurodegeneration, cancer, and infectious diseases [35].

### Mitophagy

Mitophagy is a selective autophagy process responsible for clearing damaged mitochondria, thereby maintaining cellular homeostasis and energy balance. Mitochondrial dysfunction can lead to excessive production of reactive oxygen species (ROS), apoptosis, and disease progression. The mitophagy process involves several key steps: oxidative stress or reduced mitochondrial membrane potential activates PINK1 (PTEN-inducing kinase 1) on the outer mitochondrial membrane [7, 36]. This triggers Parkin, an E3 ubiquitin ligase, which ubiquitinates mitochondrial proteins [37, 38]. These ubiquitinated proteins are then recognized by LC3 (microtubule-associated protein 1 A/1B light chain 3), facilitating mitochondrial encapsulation into autophagic vesicles [39]. Finally, these vesicles fuse with lysosomes, where mitochondria are degraded into reusable molecular components [40].

The PINK1-Parkin pathway is the most studied mitophagy mechanisms. Loss of mitochondrial membrane potential causes PINK1 accumulation, which activates Parkin to ubiquitinate mitochondrial outer membrane proteins and recruits autophagy receptor (such as p62/SQSTM1, OPTN, and NDP52), initiating autophagosome formation [36, 41]. Some mitochondrial outer membrane proteins, such as BNIP3 and NIX/FUNDC1, can also act as autophagy receptors, binding directly to LC3 to mediate mitophagy, especially under hypoxia or energy stress [42].

Mitophagy is regulated at multiple levels. AMPK and mechanistic target of rapamycin complex 1 (mTORC1) dynamically balance mitophagy by promoting or inhibiting autophagy, respectively. During cerebral ischemia, AMPK activates ULK1, while rapamycin enhances mitophagy by inhibiting mTORC1 [43, 44]. ROS accumulation damages mitochondrial DNA, triggering PINK1 accumulation Parkin recruitment [45]. Notably, peroxynitrite in brain injury enhances this pathway’s specificity, preventing the clearance of healthy mitochondria [46]. TFEB, a key transcription factor, promotes lysosomal gene expression (such as LC3 and LAMP1) [47], while FOXO3 induces BNIP3 expression under oxidative stress, promoting mitochondrial membrane depolarization [48]. The NRF2/HO- 1 axis regulates p62 expression, influencing selective autophagy [49].

Post-translational modifications, such as ubiquitination, phosphorylation, and acetylation, also fine-tune mitophagy. Parkin-mediated ubiquitination of mitochondrial proteins (such as VDAC1 and MFN2) recruits adapter proteins like OPTN/NDP52 [37, 50]. PINK1 phosphorylates ubiquitin (Ser65) and Parkin, initiating ubiquitin chain elongation, while SIRT3 activates LC3-II through deacetylation enhancing mitophagosome formation [51, 52]. Environmental factors can also modulate mitophagy. Mitochondrial calcium overload inhibits autophagosome maturation via calpain-mediated ATG5 cleavage [53, 54]. Decreases in ΔΨm expose the LC3-binding domain of PHB2, triggering mitophagy, while α-ketoglutarate lowers membrane potential by inhibiting ATP synthase, indirectly activating PINK1/Parkin pathway [55].

Mitophagy regulation is spatiotemporally specific. In acute injury, the PINK1/Parkin pathway dominates rapid clearance, while in chronic neurodegeneration, TFEB-mediated transcriptional regulation plays a greater role [56]. Additionally, AMPK not only activates ULK1 to induce mitophagy but also promotes mitochondrial biogenesis via PGC- 1α, highlighting its bidirectional regulatory role [57].

## The Role and Mechanism of Mitophagy in Different Types of Brain Injury

Mitophagy, a specialized form of macroautophagy targeting mitochondria, plays a protective role in cerebral injury by clearing damaged mitochondria, preventing apoptotic signals (e.g., cytochrome *c* release), and reducing oxidative stress. It also supports energy metabolism by maintaining ATP production. Additionally, mitophagy removes mitochondria that release damage-associated molecular patterns (DAMPs), such as mitochondrial DNA, thereby suppressing NLRP3 inflammasome activation, reducing inflammation, and alleviating neuroinflammation [58]. By eliminating dysfunctional mitochondria, mitophagy mitigates ROS production [59], a major contributor to oxidative stress, thus preserving intracellular homeostasis [60, 61].

However, dysregulation of mitophagy can negatively impact brain injury progression. Excessive autophagy activity can lead to the over-clearance of mitochondria, resulting in a shortage of these organelles and exacerbating cellular energy metabolism disorders and death. Conversely, insufficient autophagy activity can cause the accumulation of damaged mitochondria, increased ROS production, and augmented oxidative stress, thereby worsening oxidative damage and cell death [62, 63].

**Fig. 1:**
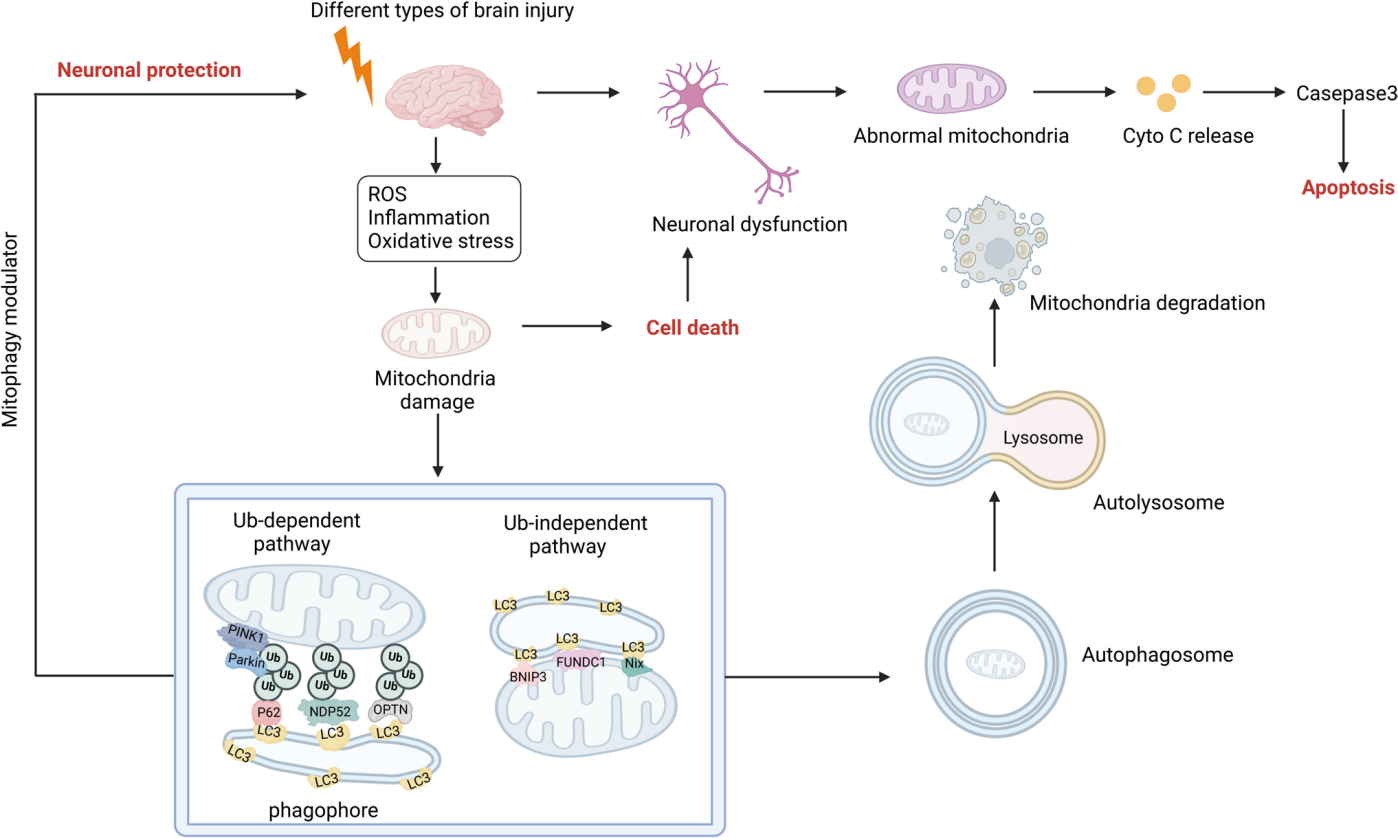
Mitophagy pathways after brain injury. Different types of brain injury activate of mitophagy in brain tissue. Mitophagy-related proteins help identify damaged mitochondria, which undergo depolarization and loss of outer membrane potential following external stimuli such as ROS. These dysfunctional mitochondria are then encapsulated by autophagosomes, forming mitochondrial autophagosomes, which later fuse with lysosomes for degradation. Mitophagy pathways are generally classified as follows: ubiquitin-dependent mitophagy—PINK1 accumulates on the outer mitochondrial membrane, leading to Parkin activation and subsequent ubiquitination of mitochondrial proteins. This recruits autophagy receptors, such as NDP52, OPTN, and p62, that interact with LC3, facilitating mitochondrial clearance. Ubiquitin-independent receptor-mediated mitophagy—autophagy receptor proteins, such as BNIP3, FUNDC1, and NIX, directly bind LC3 through their LC3 interaction region (LIR) motif, enabling autophagosome-mediated mitochondrial degradation. Brain injury leads to mitochondrial dysfunction, characterized by increased ROS production, insufficient ATP generation, and cell death, often due to insufficient mitophagy. Mitophagy modulators may protect the nervous system by enhancing mitochondrial clearance and preserving cellular function. PINK1: PTEN-induced putative kinase protein 1; NDP52: nuclear domain 10 protein 52; OPTN: NBR1 and Optineurin; p62: sequestosome- 1; LC3: microtubule-associated protein 1 light chain 3; BNIP3: BCL2 19 kDa interacting protein 3; FUNDC1: FUN14 domain-containing protein

Brain injury significantly affects health, with diverse types exhibiting varied pathogenesis and clinical manifestations. Understanding the role of mitophagy in different brain injuries aids in elucidating the molecular mechanisms underlying these conditions and offers novel insights and methods for prevention and treatment. Maintaining normal mitophagy activity is critical to protecting cells from brain injury [64]. Regulating mitophagy activity to appropriate levels helps preserve mitochondrial health within cells, thereby safeguarding against the detrimental effects of brain injury [6] (Fig. 1).

### Mitophagy in Ischemic Brain Injury

In ischemic brain injury, the interruption of blood supply deprives cells of essential oxygen and nutrients, leading to abnormal cell metabolism, energy depletion, and oxidative stress, ultimately resulting in cell death. As the primary centers of energy production within cells, mitochondria are severely affected by ischemic damage [65, 66].

Research indicates that mitophagy, initiated by ischemic brain injury, is regulated by multiple signaling pathways [67]. Under hypoxic–ischemic conditions, PINK1 is upregulated and interacts with the mitochondrial membrane protein Parkin, facilitating its attachment to the surface of damaged mitochondria [68]. This activation of Parkin leads to the ubiquitination and labeling of mitochondria, making them for packaging into autophagosomes and subsequent degradation [69]. In neonatal hypoxic–ischemic (HI) brain injury, inhibition of FUNDC1-mediated mitophagy may reduce HI-induced hippocampal neuronal damage and improve long-term cognitive function [70]. Additionally, other key molecules such as BNIP3/NIX also play a role in regulating mitophagy in ischemic brain injury [65, 71].

The process of mitophagy within cells is influenced not only by specific signaling pathways but also by various regulatory factors. It has been demonstrated that mitophagy activity under hypoxic–ischemic conditions may be regulated by protein modifications, including phosphorylation and acetylation [72]. The regulatory mechanisms of mitophagy in ischemic brain injury are complex, involving a variety of signaling pathways and regulators. These studies provide important insights into the pathogenesis of cerebral ischemic injury and offer a new perspective for the development of targeted treatment strategies in the future.

### Mitophagy in Intracranial Hemorrhagic Brain Injury

Intracranial hemorrhage refers to bleeding in the brain or surrounding tissue and is common in a variety of neurological diseases. These hemorrhagic events are highly lethal and can result in severe long-term neurological deficits [73, 74]. The main types of intracranial hemorrhage discussed include subarachnoid hemorrhage (SAH), intracerebral hemorrhage (ICH), and intraventricular hemorrhage (IVH). Although the causes and clinical manifestations of SAH, ICH, and IVH differ, they all involve complex pathophysiological processes. The role of mitophagy in ICH has received increasing attention [75]. ICH often causes severe neurological damage and high mortality. Through mitophagy, cells maintain energy balance and cellular homeostasis by clearing damaged mitochondria, thereby reducing secondary damage caused by bleeding [76, 77].

ICH and IVH are serious neurological events. These hemorrhagic events trigger a series of pathophysiological processes, including blood–brain barrier disruption, inflammatory response, oxidative stress, and cell death [78]. During cerebral or ventricular hemorrhage, hemoglobin and iron ions released from ruptured red blood cells induce oxidative stress, leading to mitochondrial membrane potential loss and dysfunction, which in turn activates mitophagy [79–81]. These pathological processes are further exacerbated by mitochondrial dysfunction. Mitophagy, by clearing damaged mitochondria, plays a protective role in these complex pathological processes [82, 83].

In oxidative stress and inflammation triggered by ICH and IVH, mitophagy reduces excessive ROS production, prevents cell apoptosis, and alleviates brain tissue damage [84]. Additionally, mitophagy regulates cell metabolism, maintains energy supply, and promotes cell survival and functional recovery [85]. Studies have shown that enhancing mitophagy, such as using autophagy inducers or regulating the PINK1/Parkin pathway through gene therapy, may become a novel treatment of ICH and IVH [86, 87]. For example, autophagy inducers like FUNDC1 have shown potential in reducing brain damage and improving neurological function in animal models [88]. By regulating and enhancing mitophagy, it is possible to significantly improve the prognosis of patients with ICH and IVH, reduce long-term neurological dysfunction, and improve their quality of life.

### Mitophagy in Traumatic Brain Injury

Traumatic brain injury (TBI) is a form of brain damage caused by an external force, commonly occurring in traffic accidents, falls, and sports-related incidents. TBI can result in extensive damage to neural cells and mitochondria, leading to brain tissue dysfunction and the development of nervous system disorders [89].

Previous studies have demonstrated that mitophagy plays an important protective role in TBI [86, 90, 91]. Recent research by Zhu et al. revealed that mitophagy deficiency exacerbates inflammation, oxidative stress, and neuronal cell death after TBI. Furthermore, this study suggests new therapeutic approaches based on mitophagy induction strategies, which help reduce cell damage and improve neuronal survival by enhancing mitophagy function [91]. It was concluded that mitophagy maintains the function of neurons and alleviates the long-term effects of TBI by promoting the clearance of damaged mitochondria [90].

Moreover, studies have demonstrated that TBI is associated with an increase in mitophagy in the human brain, suggesting that this process may represent an endogenous neuroprotective mechanism directed by cardiolipin. This implies that regulating cardiolipin levels could further activate or enhance this protective mitophagy [92]. Another study explored the effects of transferring mesenchymal stem cells (MSCs) overexpressing interleukin 10 (IL- 10) in a rat model of TBI, finding that this intervention could induce mitophagy and promote neuroprotection [93], providing an experimental basis for using cell therapy to promote mitophagy.

Niu et al. investigated the influence of mitochondrial fission inhibitors on TBI, discovering that inhibiting Drp1-dependent mitochondrial fission and reducing mitophagy can exacerbate neurological damage and neuronal death after TBI. This highlights the significance of moderate mitophagy in maintaining neural cell health [94]. Harnessing mitophagy mechanisms offers potential therapeutic strategies to mitigate the adverse effects of traumatic brain injury. Depending on mitophagy’s protective or detrimental role in TBI, various drug candidates have been identified as mitophagy promoters or apoptosis inhibitors. However, significant challenges remain in translating these findings into clinical applications, requiring further research and validation.

### Mitophagy in Neurodegenerative Diseases

Neurodegenerative diseases are a group of disorders characterized by progressive loss of neurons and functional decline [95]. Studies have demonstrated that the number of mitochondria in the brain tissue of patients with Alzheimer’s disease (AD) and Parkinson’s disease (PD) is reduced, and mitochondrial morphology is abnormal, including fragmentation, swelling, and deformation [96]. These changes indicate that the altered number and morphology of mitochondria impair their function, preventing them from effectively maintaining normal nerve cell function. Mitophagy is crucial for clearing damaged mitochondria and reducing mitochondria-induced oxidative stress and cell damage, thereby alleviating neurodegenerative changes and protecting neurological function [97, 98].

In neurodegenerative diseases, the disruption of mitophagy contributes to cell dysfunction and death [99, 100]. The regulatory mechanism of mitophagy involves multiple signaling pathways and regulatory factors [101–103]. For instance, abnormal activation of the PINK1/Parkin pathway is associated with mitophagy imbalance in AD [104]. Additionally, the dysfunction of mitochondrial proteins PINK1 and Parkin can lead to mitophagy impairment in PD [105]. Moreover, certain miRNAs related to neurodegenerative diseases, such as miR- 34a and miR- 181c, have been found to regulate mitophagy [106].

These findings suggest that enhancing mitophagy could offer novel therapeutic strategies for currently untreatable neurodegenerative diseases. By improving mitophagy, the disease process may be slowed, potentially improving the quality of life of patients and opening new avenues for treatment [107, 108]. However, despite preliminary confirmation of mitophagy’s role in these diseases, further research and validation are needed to fully understand its regulatory mechanisms and develop effective therapeutic strategies.

### Mitophagy in Radiation-Induced Brain Injury

Radiation-induced damage to mitochondria and mitochondrial DNA impairs mitochondrial function and triggers oxidative stress, which in turn initiates inflammatory responses and affects mitochondrial structure and function [109–111]. If these damaged mitochondria are not cleared promptly, they can cause a series of intracellular signaling pathway disorders, exacerbating brain damage [112, 113].

Studies have demonstrated that the activity of the PINK1/Parkin pathway may be influenced by radiation’s adverse effects on the brain [114]. On one hand, radiation can activate the PINK1/Parkin pathway, thereby enhancing mitophagy efficiency [115]. On the other hand, radiation may also cause abnormal expression of other molecules in the pathway, leading to inhibition of mitophagy [116]. Defective mitophagy has been demonstrated to exacerbate neuroinflammation, oxidative stress, and cell death, negatively impacting the development of radiation-induced brain injury [94, 112, 117]. Therefore, regulating mitophagy activity may emerge as a novel approach to treating radiation-induced brain injury.

Abnormal mitophagy due to radiation brain injury can exacerbate cell damage and neuroinflammatory responses, impairing the normal function of brain tissue. Modulation of the PINK1/Parkin pathway may represent a novel therapeutic avenue for radiation-induced brain injury (RIBI) treatment, potentially protecting the stability of the central nervous system by reducing cell death and oxidative stress through enhanced mitophagy efficiency [115]. It is hypothesized that mitophagy enhancement, particularly in mitigating radiation-induced DNA damage, could be a key strategy for RIBI prevention. Further investigation is required to identify other molecular mechanisms that may affect mitophagy and to develop targeted therapeutic strategies for treating RIBI.

### Application of Mitophagy Regulation in the Treatment of Brain Injury

Extensive research has been conducted on treating brain injury, with a particular emphasis on the potential of different drugs to reduce brain injury by regulating mitophagy. Table 1 summarizes various therapeutic agents and their mechanisms of action across different types of brain injuries, including ischemic brain injury, intracranial hemorrhagic disease, TBI, neurodegenerative diseases, and RIBI. The table also categorizes these agents into agonists and inhibitors, highlighting their specific mechanisms and effects on injury outcomes, such as reducing neuronal apoptosis, alleviating oxidative stress, and improving neurological functions.

**Table 1:** The molecular mechanisms and neuro-effects of mitophagy modulators in brain injuries

Brain injury	Agonist	Inhibitor	Mechanism	Effect on brain injury	Ref
Ischemic brain injury	DHA		PINK1/Parkin	Protected against neuronal apoptosis	[118]
	Ori		AMPK	Ameliorated caspase- 9-mediated brain neuronal apoptosis	[119]
	UMB		Parkin-dependent manner	Protected against cerebral ischemic injury	[120]
	Salidroside		AMPK	Promoted mitochondrial biogenesis in neurons	[121]
	tPA		Increase the phosphorylation of AMPK and the expression of FUNDC1	Inhibited apoptosis and improved mitochondrial function	[122]
	Garciesculenxanthone B		PINK1/Parkin	Neuroprotective effect	[123]
	AFPR		PINK1/Parkin	Reduced neurological scores and infarct size, alleviated neuron apoptosis in cortex	[124]
		lncRNA TUG1	SIRT1-induced mitophagy	Relieved the neuronal apoptosis	[125]
	Xuesaitong combined with dexmedetomidine		PINK1/Parkin	Ameliorated oxidative stress and mitochondrial dysfunction	[126]
Intracranial hemorrhagic brain injury	T3		PINK1/Parkin	Decreased microglial activation, alleviated neuroinflammation	[127]
	Metformin		AMPK	Attenuated neuronal apoptosis	[128]
	NL- 1		PINK1/Parkin	Reduced oxidative stress and apoptosis	[129]
	Mdivi- 1		FUNDC1/NLRP3	Inhibit NLRP3 inflammasome activation	[88]
	SENP1		SENP1-mediated OPTN deSUMOylation	Promoted neuronal differentiation of OM-MSCs through OPTN-mediated mitophagy to improve neurological deficits	[84]
TBI	IGF- 1		NF-κB pathway transcriptionally regulates decapping mRNA2 and miR-let- 7	Decreased neuron cell death, suppressed apoptosis, pyroptosis, and ferroptosis	[130]
	HucMSC-derived exosome		PINK1/Parkin	Improved neurological function, decreased cerebral edema, and attenuate brain lesion	[131]
Neurodegeneration disease	Quercetin		Inhibition NLRP3 inflammasome activation	Alleviated neurotoxicity	[132]
	Amyloid β1–40		CD36/PINK1/Parkin	Alleviated BBB disruption	[133]
	Pramipexole (PPX)		BNIP3/PINK1/Parkin	Mitigated neuronal injury	[134]
	Formoterol		PINK1/Parkin and PI3 K/Akt/CREB/BDNF/TrKB axis	Mitigated neuro-inflammatory status	[135]
RIBI	Vinpocetine (VIN) and coenzyme Q10 (CoQ10)		PINK1/Parkin	Mitigated cognitive dysfunction, neurons loss, mitochondrial damage, and oxidative stress injury	[136]

*TBI* traumatic brain injury, *SAH* subarachnoid hemorrhage, *RIBI* radiation-induced brain injury, *IGF- 1* insulin-like growth factor- 1, *HucMSC* human umbilical cord mesenchymal stem cell, *DHA* docosahexaenoic acid, *Ori* oridonin, *UMB* umbelliferone, *T3* triiodothyronine; *tPA* tissue-type plasminogen activator, *AFPR* active fraction of *Polyrhachis vicina*, *TUG1* taurine upregulated gene 1, *PPX* pramipexole, *MMP- 9* metalloproteinase 9, *VIN* vinpocetine, *CoQ10* coenzyme Q10, *SENP1* SUMO-specific protease 1, *OPTN* Optineurin

For cerebral ischemic brain injury, compounds such as eicosahexaenoic acid (DHA), podophyllin (Ori), imperatorin (UMB), tissue plasminogen activator (tPA), garciesculenxanthone B, active fraction of *Polyrhachis vicina* (AFPR), xuesaiton (XST) combined with dextromethamine Tomidine (Dex), salidroside, and lncRNA Taurine upregulated gene 1 (TUG1) have been shown to inhibit apoptosis, improve hippocampal neuron damage, enhance mitochondrial function, and alleviate oxidative stress. These effects are achieved by regulating pathways like PINK1/Parkin, AMPK, ULK1, SIRT1, BNIP3, and Drp1, which are associated with mitophagy and mitochondrial dynamics [118–126].

In the treatment of ICH, thyroxine (T3), NL- 1, metformin, Mdivi- 1, and SENP1 (SUMO-specific protease 1) have been found to reduce microglial activation, neuroinflammation, oxidative stress, and apoptosis. These benefits occur through pathways including PINK1/Parkin, AMPK, FUNDC1/NLRP3, or OPTN (Optineurin)/deSUMOylation [84, 88, 127–129].

In the context of TBI, studies have identified that insulin-like growth factor- 1 (IGF- 1) and human umbilical cord mesenchymal stem cells (HucMSC) enhance the expression of mitophagy markers through the exosome pathway. This improvement in neurological function reduces neuronal cell death and inhibits apoptosis, pyroptosis, and ferroptosis [130, 131].

In neurodegenerative diseases, quercetin, amyloid beta (Aβ)− 40, pramipexole (PPX), and formoterol have been shown to inhibit the NLRP3 inflammasome, reduce neurotoxicity and neuroinflammatory, improve mitochondrial function, and maintain blood–brain barrier integrity. These effects are mediated by regulating the PINK1/Parkin, PI3 K/Akt, and CREB/BDNF pathways [132–135].

**Fig. 2:**
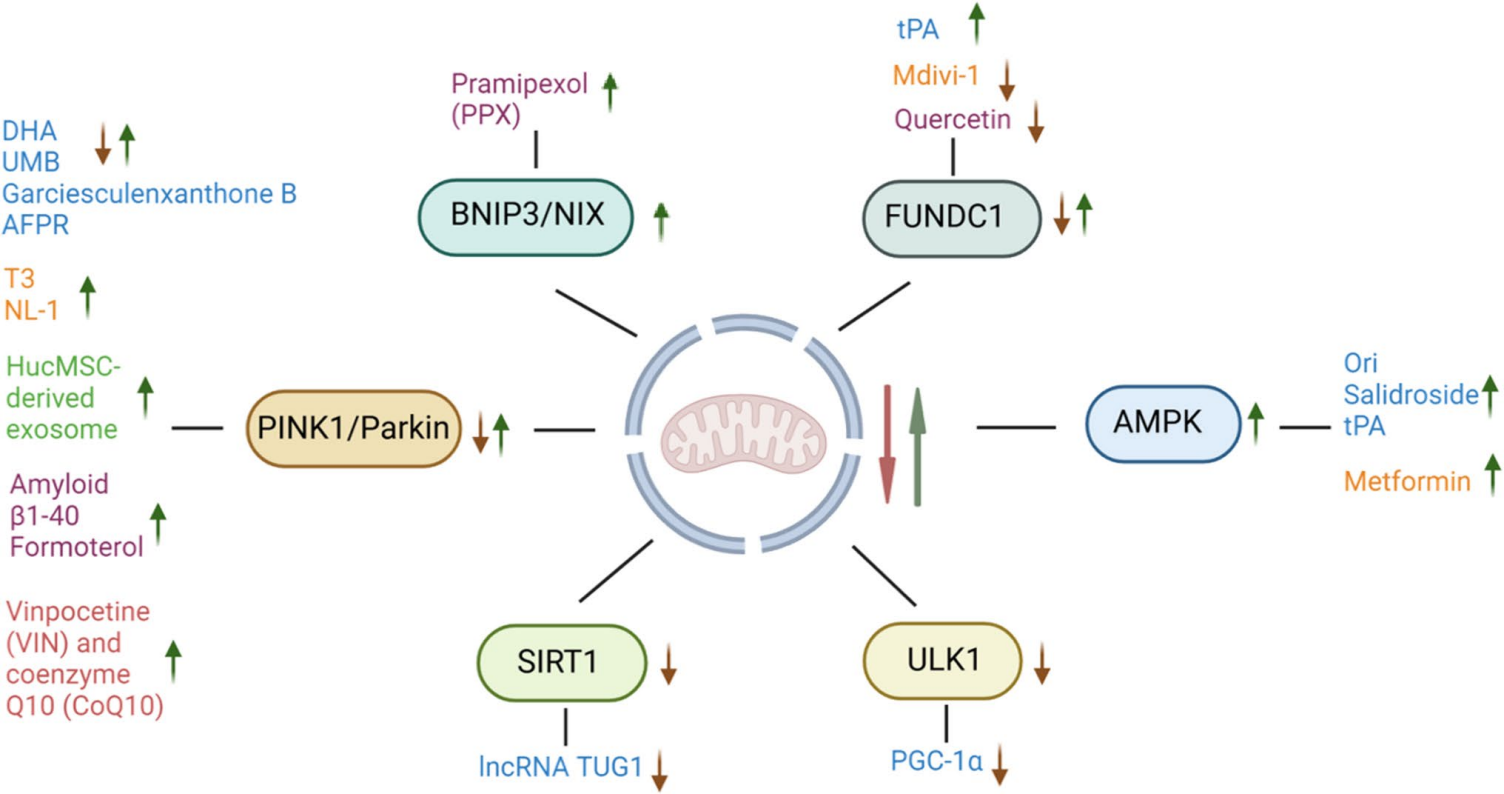
Mitophagy regulators and their targets. Mitophagy is regulated by multiple molecular pathways that influence brain injury outcomes. Upregulation of PINK1/Parkin, BNIP3/NIX, FUNDC1, and AMPK enhances mitophagy, promoting neuroprotection and mitigating brain damage. Conversely, downregulation of ULK1 and SIRT1 reduces mitophagy, exacerbating cellular damage and worsening injury

For RIBI, vinpocetine (VIN) and coenzyme Q10 (CoQ10) have demonstrated potential in alleviating cognitive dysfunction, reducing neuronal loss, and mitigating mitochondrial damage and oxidative stress by assessing mitophagy in hippocampal neurons [136]. These preclinical studies highlight the potential of various drugs in reducing brain injury by regulating the mitophagy pathway, offering significant insights for future clinical treatments (Fig. 2).

Therapeutic strategies targeting mitophagy may exhibit synergistic effects when combined with other treatment modalities. Experimental evidence suggests that overexpression of the copper chaperone antioxidant 1 (Atox1) provides neuroprotection against TBI-induced cognitive deficits by DJ- 1-dependent antioxidative pathways and enhancing mitophagy [137].

Quercetin (Qu), a flavonoid with anti-inflammatory and antioxidant properties, mitigates neural damage by enhancing mitophagic clearance, reducing mitochondrial ROS (mtROS) accumulation, and suppressing NLRP3 inflammasome activation [132]. In AD models, the NLRP3 inhibitor MCC950 alleviates cognitive deficits and anxiety-like behaviors through dual mechanisms of neuroinflammatory suppression and autophagy regulation [138].

Bone marrow–derived mesenchymal stem cells (BMSCs) provide neuroprotection in cerebral ischemia by regulating antioxidant defenses and mitochondrial quality control. They upregulate glutathione (GSH) to neutralize ROS while activating selective mitophagy pathways to eliminate dysfunctional mitochondria and inhibit apoptosis. This dual action reduces neuronal apoptosis and promotes functional recovery post-ischemic stroke, offering promise for cell-based therapies in cerebrovascular disorders. [139]. The convergence of these mechanisms highlights the therapeutic potential of combining mitophagy modulation with antioxidant strategies for neurological disease treatment.

## Concluding Remarks

Recent advances in mitophagy research highlight its crucial role in both pediatric and adult brain injury. By clearing damaged mitochondria, mitophagy reduces cell death and promote brain tissue repair [91, 140]. However, its regulatory mechanisms vary by injury type [114, 141–143] and are influenced by factors, such as age, gender, and genetics, necessitating further investigation [144]. Combining mitophagy targeted therapies with other therapeutic modalities may enhance neuroprotection through synergistic effects. Understanding the complex neuroprotective network centered on mitophagy and its underlying mechanisms is crucial for optimizing therapeutic strategies.

To develop targeted treatments, a deeper understanding of mitophagy’s regulatory mechanism across different brain injury is essential [145]. Clinical research should focus on evaluating the effects of drugs on mitophagy through trials, which will facilitate advance brain injury treatments. Future research should also address individual differences in mitophagy to achieve personalized treatment, improving therapeutic outcomes and patient quality of life. Understanding the influence of age, gender, and genetic background on mitophagy can lead to more effective, individualized strategies for brain injury.

## Data Availability

No datasets were generated or analysed during the current study.
